# Synthesis of AgInS_2_-*x*Ag_2_S-*y*ZnS-*z*In_6_S_7_ (*x*, *y*, *z* = 0, or 1) Nanocomposites with Composition-Dependent Activity towards Solar Hydrogen Evolution

**DOI:** 10.3390/ma9050329

**Published:** 2016-04-29

**Authors:** Zhaojie Wang, Shutao Wang, Junxue Liu, Wen Jiang, Yan Zhou, Changhua An, Jun Zhang

**Affiliations:** 1 College of Science, China University of Petroleum, Qingdao 266580, China; wangzhaojie@upc.edu.cn (Z.W.); shtwang@upc.edu.cn (S.W.); liujx@sina.cn (J.L.); yanzhou@upc.edu.cn (Y.Z.); 2 State Key Laboratory of Heavy Oil Processing, College of Chemical Engineering, China University of Petroleum, Qingdao 266580, China; jiangwenS110@163.com

**Keywords:** heterojunction, AgInS_2_-*x*Ag_2_S-*y*ZnS-*z*In_6_S_7_, photocatalysis, H_2_ evolution

## Abstract

Metal sulfides-based nanomaterials have been used as a class of efficient solar driven photocatalysts. However, the H_2_-production rate observed over these photocatalysts remains problematic. Here, the AgInS_2_-*x*Ag_2_S-*y*ZnS-*z*In_6_S_7_ (*x*, *y*, *z* = 0 or 1) nanocomposites with controlled compositions have been successfully prepared by a simple hydrothermal method with AgI polyhedrons as silver source. The obtained AgInS_2_-*x*Ag_2_S-*y*ZnS-*z*In_6_S_7_ nanocomposites showed a composition-dependent activity for H_2_ evolution from aqueous solution under simulated sun-light irradiation. The results showed that the optimized product of AgInS_2_-Ag_2_S-ZnS nanoparticles synthesized with the precursor ratio of Ag:Zn = 1:1 exhibited the highest H_2_ evolution rate of 5.4 mmol·g^−1^·h^−1^. Furthermore, the catalyst can be used for 20 h without loss of activity, showing its high stability. It opens a new path to achieve highly efficient solar photocatalyst for H_2_ evolution from water splitting.

## 1. Introduction

Two major kinds of photocatalysts in terms of composition are mainly used to drive solar water-splitting [1]. One is single-component material possessing proper band structure aligned for hydrogen generation. The other is multi-component composite made of different photo-absorbers with the matched band alignments to split water. The bandgap of these materials should be relatively narrow, which can efficiently favor the absorption of solar light [2,3,4]. Additionally, the conduction band edge must be sufficiently more negative than the reduction potential of protons. Compared with single-component material, making a complex catalyst composite has several advantages. First, the potentials of the conduction and valence bands shift successively with composition. According to the band gap transition, the composite is able to absorb enough visible light. Second, photogenerated electrons and holes can move smoothly in the continuous valence and conduction bands, rather than in the discrete levels seen in the single photocatalyst. A series of ZnS-CuInS_2_-AgInS_2_ with a different percentage of the components have been successfully prepared, whose absorption edges were shifted monotonically to longer wavelengths as the ratio of MInS_2_ (M = Cu and Ag) to ZnS increases [5]. Moreover, optoelectronic materials with electronically coupled components usually possess interesting and unique optical and electronic properties, *i.e.*, enlarged absorption region of the solar spectrum, facilitated charge transportation without increasing the rate of charge recombination, and enhanced charge collection efficiency [6,7]. These excellent properties make optoelectronic composites potential candidates for application in the fields of photocatalysis. The nanocomposite theoretically has the ability to provide a high photocatalytic water-splitting efficiency. The heterojunction with different energy levels may form an ideal system to cause a rapid photo-induced charge separation and decreased recombination chance of electron–hole pairs [8,9]. Therefore, under the irradiation of solar energy, a photoinduced electron would transfer efficiently in photochemically stable semiconductor nanocrystals by the synergetic effect of the multi-component system [10,11,12,13].

As is the case with metal oxide semiconductors, e.g., TiO_2_ and ZnO, I-III-VI_2_ (e.g., (Cu)AgInS_2_) [14,15,16,17,18] and II-VI (e.g., Zn(Cd)S) [19,20,21,22], groups have been developed as a new platform for photocatalytic applications. With the rapid growth of research in this area, synthesis strategies such as alloying, doping, and solid solution nanomaterials have attracted much attention with regard to band gap and electronic wave function engineering [23,24]. For example, Kudo and Peng synthesized (AgIn)*_x_*Zn_2(1-*x*)_S_2_ and (CuIn)*_x_*Zn_2(1-*x*)_S_2_ solid solutions with the capacity to reduce water to H_2_ under visible light [14,17]. Mao *et al.* investigated the tunable light absorption and photoluminescence properties of AgInS_2_/ZnS nanocrystals [25]. Michalska *et al.* synthesized core/shell CuInS_2_/ZnS quantum dots and studied the optical features and structure in detail [26]. However, there are no obvious heterointerfaces in solid solutions and the doping system, and it is difficult to achieve the morphology and size control. In semiconductor nanocomposites, alloying of the heterojunction interface can reduce the lattice mismatch between different components, where the mixed compositions allow for gradual strain release with improved optical properties [27,28]. The reaction chemistry of the AgInS_2_-ZnS system is complicated and very intriguing. It can favor the design of photocatalysts with widely tunable properties. However, it is still not well understood how to control the structure of the multi-component, and to tune the photochemistry properties of alloying semiconductors.

Herein, we have developed a facile hydrothermal method to prepare AgInS_2_-*x*Ag_2_S-*y*ZnS-*z*In_6_S_7_ (*x*, *y*, *z* = 0 or 1) nanocomposites. The compositions of the resulting nanocomposites can be controlled through varying the ratios of feeding stocks, which are the main factors in determining their activities towards solar hydrogen evolution. The experimental results demonstrated that the heterojunction structures exhibited much higher photocatalytic activity than single AgInS_2_ and AgInS_2_-Ag_2_S for solar H_2_ evolution.

## 2. Results and Discussion

**Figure 1:**
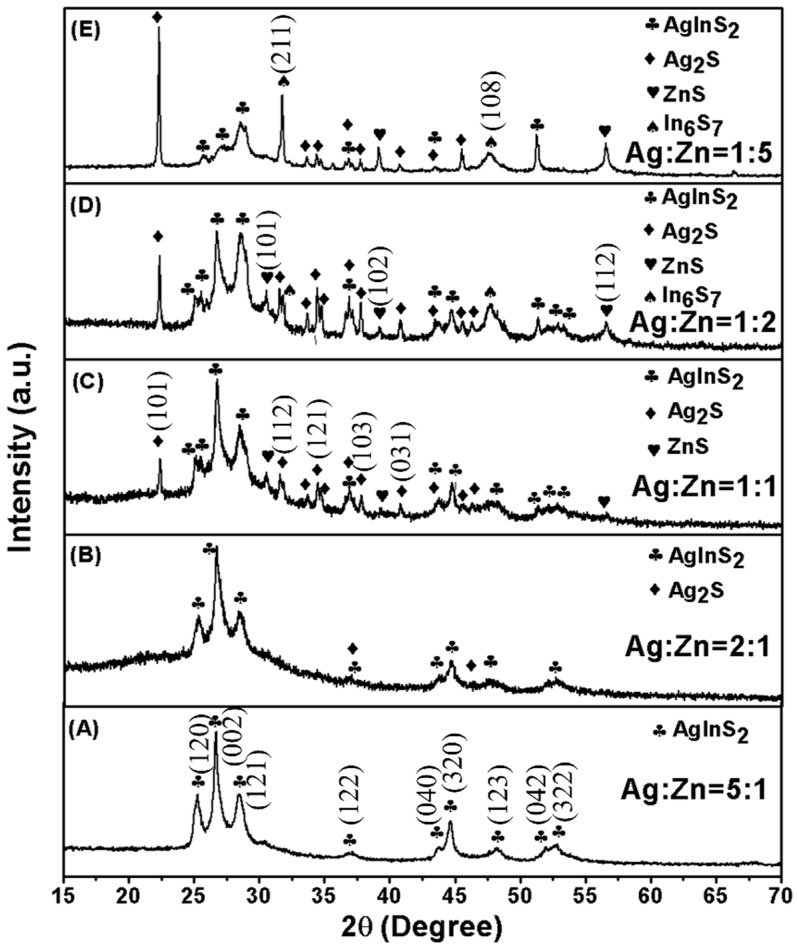
X-ray diffraction patterns of the as-synthesized AgInS_2_-*x*Ag_2_S-*y*ZnS-*z*In_6_S_7_ nanojunctions with different molar ratios of Ag to Zn. AgInS_2_: JCPDS 25-1328, Ag_2_S JCPDS 14-0072, ZnS: JCPDS 36-1450, In_6_S_7_: JCPDS 19-0587.

In order to obtain an optimized efficient solar photocatalyst for H_2_ production, the multi-component nanocomposites were synthesized at various molar ratios of Ag^+^ to Zn^2+^ ions. Figure 1 shows XRD patterns of the as-synthesized AgInS_2_-*x*Ag_2_S-*y*ZnS-*z*In_6_S_7_ nanoheterojunctions produced at different Ag:Zn ratios. It shows that only single orthorhombic phase of AgInS_2_ is obtained when the starting molar ratio of Ag:Zn is 5:1 (Figure 1A). Compared with tetragonal AgInS_2_, the orthorhombic AgInS_2_ is usually crystallized at high temperature. In our work, the metastable orthorhombic AgInS_2_ can be obtained under a mild condition (180 °C), which may be due to the proper chemical environment and conditions used for the materials preparation. With decreasing the ratio to 2:1, monoclinic Ag_2_S appears in the product (Figure 1B). Further increasing the dosage of Zn^2+^ (Ag:Zn = 1:1), as shown in Figure 1C, the characteristic peak of hexagonal ZnS appears, meaning that AgInS_2_-Ag_2_S-ZnS nanocomposite formed. However, as shown in Figure 1D,E, further increasing of the amount of Zn^2+^ ions it results in the formation of In_6_S_7_, and the product thus switches to AgInS_2_-Ag_2_S-ZnS-In_6_S_7_. It is clear that no other characteristic peaks for external impurities are observed, indicating that the phase composition of the final products can be controlled through varying the addition of Zn^2+^ ions.

**Figure 2:**
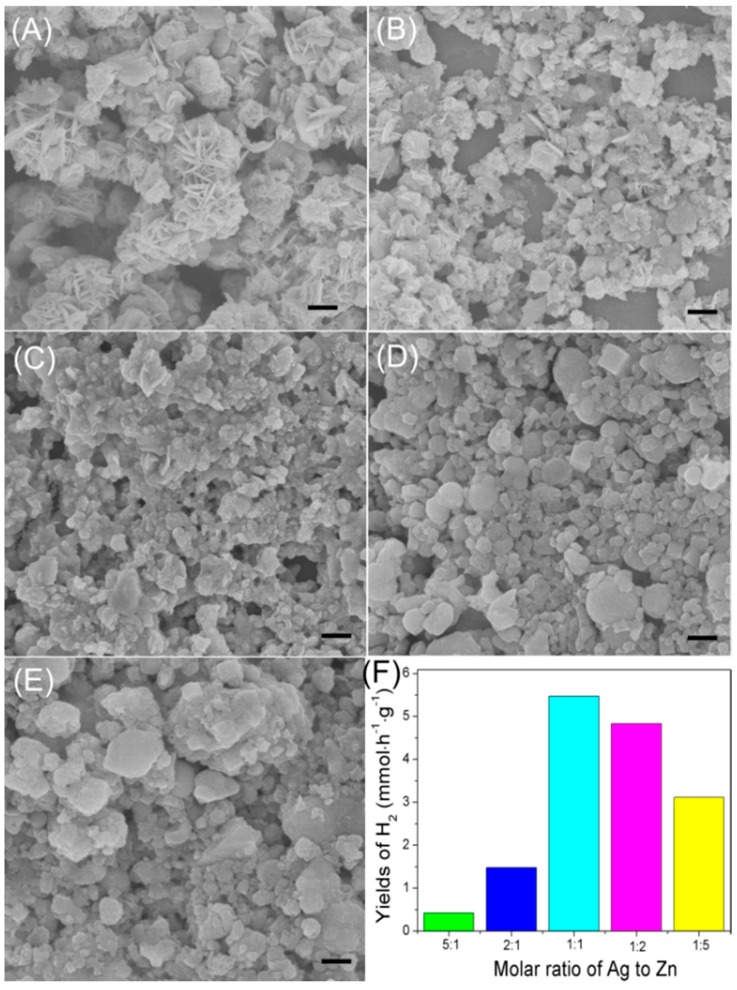
SEM images of AgInS_2_-*x*Ag_2_S-*y*ZnS-*z*In_6_S_7_ composite nanoparticles with different molar ratios of Ag:Zn (**A**) 5:1; (**B**) 2:1; (**C**) 1:1; (**D**) 1:2; (**E**) 1:5 (The scale bars are all 400 nm); and a corresponding comparison chart of the photocatalytic H_2_ production in a reaction period of 12 h (**F**).

Figure 2 shows a series of SEM images of the as-synthesized samples and a comparison of their solar hydrogen evolution property correspondingly. As revealed in Figure 2A–E, different molar ratios of Ag:Zn result in the formation of products with diverse compositions and morphologies. The obtained AgInS_2_ nanoparticles with molar ratio of Ag:Zn at 5:1 display regular flowers with the size of 810 nm and are assembled by many nanosheets (Figure 2A). With the ratio decreased to 2:1, the produced AgInS_2_-Ag_2_S is mainly composed of nanoplates with a length of 300–400 nm and thickness of several tens of nanometer (Figure 2B). However, further increasing the dosage of zinc ions, *i.e.*, Ag:Zn = 1:1, as shown in Figure 2C, the as-obtained AgInS_2_-Ag_2_S-ZnS shows aggregation of the nanoplates. When the mole number of Zn is higher than that of Ag, the product contains AgInS_2_-Ag_2_S-ZnS-In_6_S_7_. The particles reveal irregular and aggregated morphology (Figure 2D,E). Figure S1 shows diffuse reflectance spectra (DRS) of the prepared composites. Obviously, when the amount of Zn added in the preparation is higher than that of Ag (Ag:Zn = 1:5 or 1:2), the DRS spectra exhibit intense absorption bands with steep edges, which mainly depends on the UV absorption band of ZnS. With the decrease in the dosage of Zn(Ac)_2_, the composites have absorptions in the range of visible light (λ > 450 nm), whereas ZnS has little absorption in this range. The presence of AgInS_2_ and Ag_2_S contributes to the increased visible-light absorption. Notably, the absorption spectrum of AgInS_2_-*x*Ag_2_S-*y*ZnS-*z*In_6_S_7_ composite photocatalysts can be easily controlled by modifying the dosage of Zn(Ac)_2_ continuously. The complexity of the composite in both structure and chemical composition may endow the materials with exceptional optical properties.

Figure 2F shows the photocatalytic activities of the photocatalysts. The hydrogen production mainly depends on the compositions regardless of their morphology. The AgInS_2_-Ag_2_S-ZnS with the feed ratio of Ag:Zn at 1:1 exhibits the highest activity with a hydrogen evolution rate of 5.4 mmol·g^−1^·h^−1^, which is higher than that of previous reported AgInS_2_, Ag_2_S, ZnS composite photoctalysts (comparison results is shown in Table S1). Moreover, the photocatalyst presents good stability even after 20 h duration (Supplementary Materials, Figure S2). The pristine AgInS_2_ nanoparticles possessed very weak photo-activity towards H_2_ evolution. Of course, Pt salt added in the photocatalytic reaction played an important role in promoting the photocatalytic activity. Without cocatalyst of Pt salt, the catalyst itself also achieved a comparable activity towards solar hydrogen evolution of a rate of 1.8 mmol·g^−1^·h^−1^ (Supplementary Materials, Figure S3). Figure S4 reveals the surface area of the samples, suggesting that surface characteristics do not dominate the photocatalytic activity for H_2_ evolution. In other words, activities of the nanocomposites are mainly determined by their compositions.

**Figure 3:**
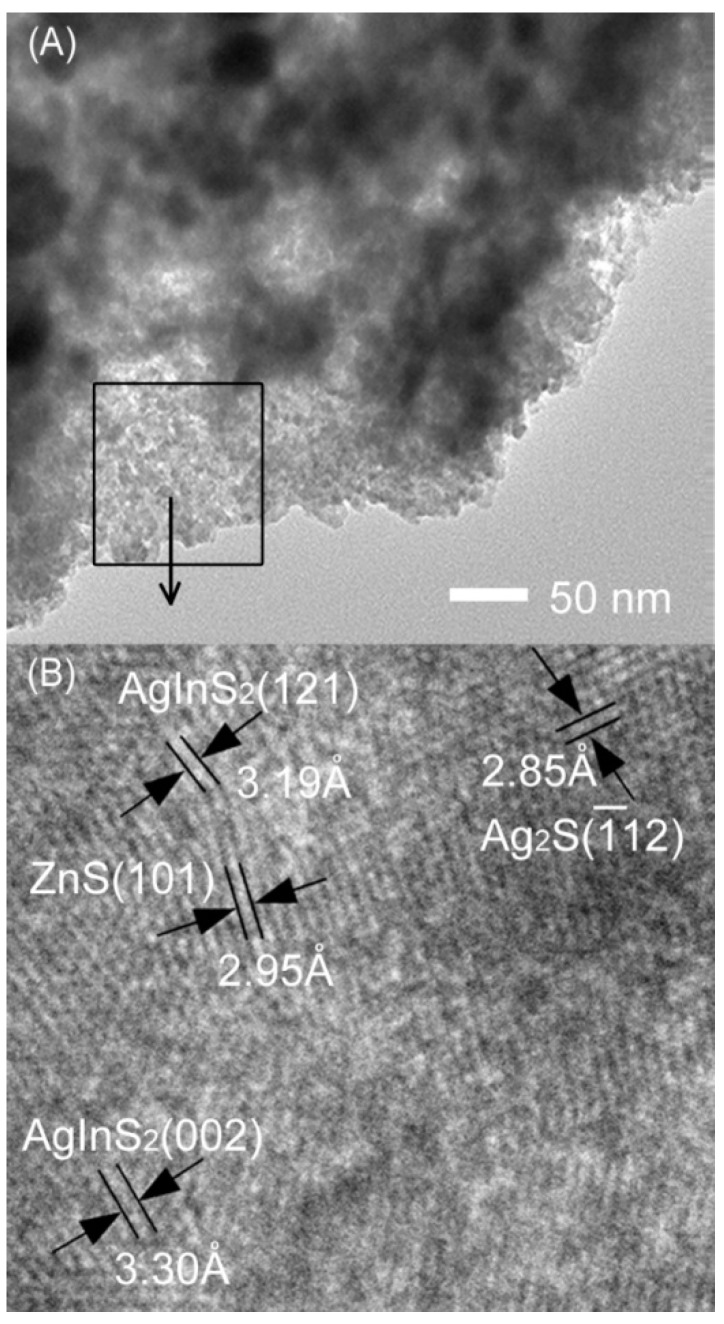
Transmission electron micrograph (TEM) (**A**) and HRTEM (**B**) images of the AgInS_2_-Ag_2_S-ZnS composite nanoparticles.

Typically, we further investigated the detailed structures of AgInS_2_-Ag_2_S-ZnS with the maximum activity by HRTEM measurement. Figure 3 clearly shows that the AgInS_2_-Ag_2_S-ZnS nanocomposite has clear interconnecting lattice fringes with interplaner spacing distance of 0.319, 0.285, and 0.295 nm, which correspond to (121) and (002) planes for orthorhombic AgInS_2_, (−112) plane for monoclinic Ag_2_S, and (101) plane for hexagonal ZnS, respectively. The results demonstrate that the photocatalyst composites have been successfully prepared through the simple hydrothermal method.

**Figure 4:**
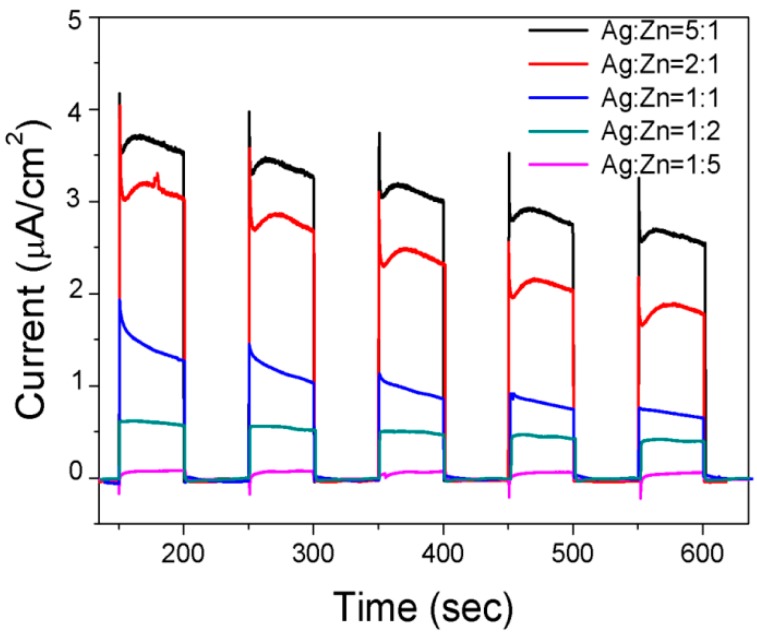
Photocurrent-density responses (I-t) of the samples synthesized with different ratios of Ag:Zn in 0.5 M Na_2_SO_3_ solution under simulated sunlight illumination.

The photocurrent response is also used to compare the photo-activity on the as-synthesized samples. As shown in Figure 4, all the samples can be excited to produce photo-carriers under simulated sunlight illumination. The electrons transport from the photocatalysts to ITO glass surface and then external circuit to produce photocurrent [29]. Prompt and reproducible current responses during repeating on-off illumination cycles are observed from each electrode of the five samples. When the illumination stops, the photocurrent drops to zero immediately. The photocurrent density increases with the increase of ratio of Ag:Zn. For AgInS_2_-Ag_2_S-ZnS-In_6_S_7_ (Ag:Zn = 1:5), the photocurrent response is very weak. The result can be ascribed to the increased content of ZnS bearing a wide bandgap (3.55 eV), leading to the low utilization ratio of solar photons. In contrast, the AgInS_2_-Ag_2_S-ZnS-In_6_S_7_ produced at Ag:Zn = 1:2 exhibits an improved photocurrent. For the AgInS_2_-Ag_2_S-ZnS obtained at Ag:Zn = 1:1, a photocurrent peak was observed at the initial irradiation stage, followed by a slow decrease before reaching a constant value. It suggests that recombination of photo-generated electrons and holes occur in the process [30]. Meanwhile, the photocurrent of the other two samples obtained with Ag:Zn = 2:1 and Ag:Zn = 5:1, *i.e.*, AgInS_2_-ZnS and AgInS_2_, are significantly higher than the above samples. Nevertheless, the current decreases along with the irradiation time, indicating the easy excitation and fast recombination of photo-generated carriers. The results are in accordance with previous reports [31], which suggest that the production of photocurrent is primarily determined by the transporting speed of excited electrons from semiconductor to ITO and the recombination at the electrolyte/film interface. Considering the photo-activity of photocurrent production and H_2_ evolution, it is deduced that the photocatalysts with stable and high photocurrent are favorable for the H_2_ generation. Therefore, the production and transfer rate of photo-generated electrons for the samples obtained with Ag:Zn of 1:1, 1:2, 1:5 are consisted with their hydrogen evolution activity. For the AgInS_2_ and AgInS_2_-ZnS nanoparticles obtained from Ag:Zn of 5:1 and 2:1, the hydrogen evolution rates are lower although their photocurrents are higher than the others.

**Figure 5:**
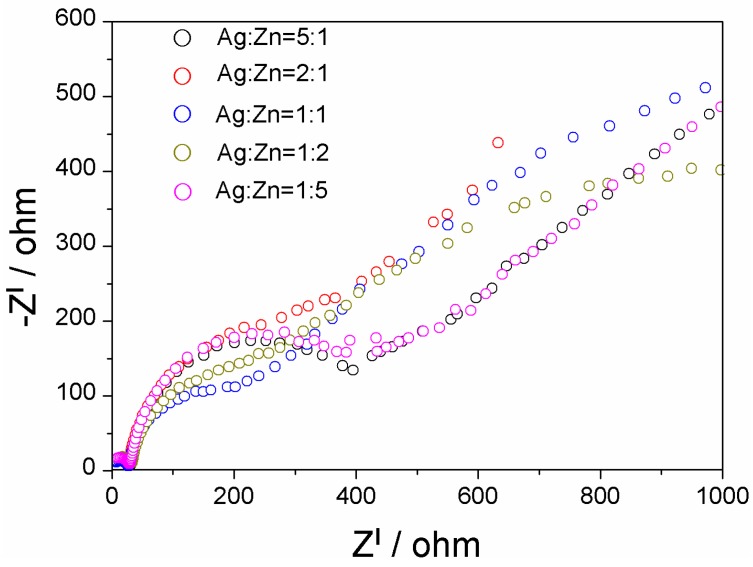
Nyquist plots of electrochemical impedance spectra (EIS) of the as-prepared samples (AgInS_2_-*x*Ag_2_S-*y*ZnS-*z*In_6_S_7_). The EIS measurements were performed in the presence of a 2.5 mM K_3_[Fe(CN)_6_]/K_4_[Fe(CN)_6_] (1:1) mixture as a red-ox probe in 0.5 M Na_2_SO_4_ aqueous solution.

Electrochemical impedance spectroscopy (EIS) is also a powerful technology to probe the charge transfer process at an electrode interface [32,33]. The semicircle at high frequencies represents the capacitance and the resistance of the solid-state interface layer, which is formed at highly charged states and results from the passivation reaction between the electrolyte and the surface of the electrode [33]. As shown in Figure 5, the semicircle diameters of AgInS_2_-Ag_2_S-ZnS (Ag:Zn = 1:1) and AgInS_2_-Ag_2_S-ZnS-In_6_S_7_ (Ag:Zn = 1:2) are smaller than those of the other samples, The higher hydrogen evolution activities would be attributed to faster interfacial electron transfer. The moderate amounts of ZnS and In_6_S_7_ added into AgInS_2_-Ag_2_S benefit the charge transfer and lower the recombination probability of photogenerated charges. Consequently, the ZnS and In_6_S_7_ nanoparticles can work as electron acceptors and transporters in the obtained composite, which significantly enhances the photocatalytic activity for H_2_ production.

**Figure 6:**
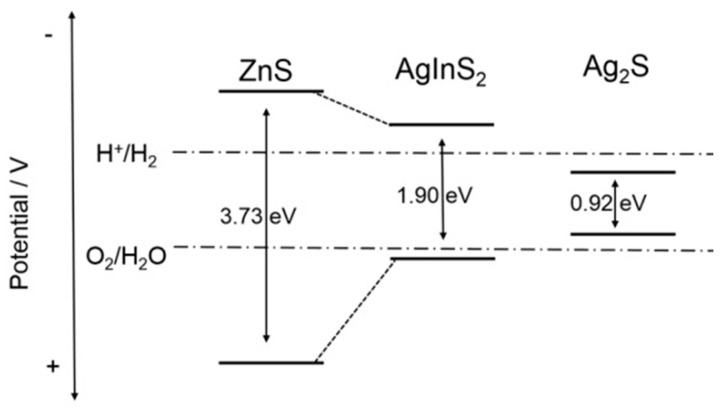
A diagrammatic scheme of energetic band structure of AgInS_2_-Ag_2_S-ZnS (Ag:Zn = 1:1).

It is known that Ag_2_S is a narrow band gap semiconductor and may have an extended absorption range, thus it can be easily excited to generate h^+^-e^−^ pairs and produce intense photo-current [34,35]. However, these generated electrons are unstable to reduce H_2_O into H_2_ due to their lower conduction band potential than H^+^/H_2_ pair. As shown in Figure 6, AgInS_2_ has an appropriate band structure for water splitting to evolve H_2_ [36]. Since the conduction band level is negative enough for a reduction potential of H_2_O/H_2_, ZnS with a wide bandgap (3.73 eV) is an ideal photocatalyst from the viewpoint of preeminent ability to generate H_2_ [37]. Herein, photocatalyst of AgInS_2_-Ag_2_S-ZnS possesses the best photocatalytic activity among the as-obtained samples with various ratios of Ag:Zn and excessive quantity of ZnS is unfavorable for solar light absorption to produce H_2_. However, regarding the feasible point of modifying by In_6_S_7_, more experimental and theoretical evidence is needed to clarify this in the future.

## 3. Materials and Methods

### 3.1. Materials

Silver acetate (AgAc), zinc acetate (Zn(Ac)_2_), potassium iodide (KI), polyvinylpyrrolidone (PVP) and ethylenediamine (En) were purchased from Sinopharm Chemical Reagent Co., Ltd. (Shanghai, China). Indium trichloride (InCl_3_∙4H_2_O), sodium diethyldithiocarbamate trihydrate ((C_2_H_5_)_2_NCSSNa∙3H_2_O, Na(DDTC)), polyethylene glycol (PEG, molecular weight = 10,000 g∙mol^−1^) were purchased from Shanghai SSS Reagent Co., Ltd. (Shanghai, China). All the reagents were analytical grade and used as received without further purification.

### 3.2. Synthesis of AgI Polyhedrons

In a typical procedure, 0.05 g of PVP and 0.06 g of AgAc were dissolved in a mixture of 10 mL of deionized water and 1 mL of En under magnetic stirring at 60 °C. After they were totally dissolved, 5 mL of KI aqueous solution (0.084 mol/L) was introduced at a rate of 1 mL/min to the above solution with a syringe pump and the resulting mixture was stirred continuously for 10 min. SEM image of the obtained AgI precursor is given in Supplementary Materials, Figure S5.

### 3.3. Synthesis of In(DDTC)_3_

Fifty millilitres of ethanol solution of 0.1 mol/L of InCl_3_∙4H_2_O and 0.3 mol/L of Na(DDTC) were mixed under vigorous magnetic stirring for ~30 min. The obtained white product was rinsed with de-ionized water 3 times and collected via vacuum filtration.

### 3.4. Synthesis of AgInS_2_-Ag_2_S-ZnS Heterojunction Nanostructures

The various nanocomposites were synthesized by a simple hydrothermal method through varying the ratios of starting materials. Briefly, the freshly synthesized AgI polyhedrons (0.12 g), 0.3 g of In(DDTC)_3_, 0.08 g of Zn(Ac)_2_ (molar ratio of Ag:Zn = 1:1) and 25 mL of deionized water were sequentially transferred into a Teflon-lined autoclave with the capacity of 45 mL. The autoclave was sealed tightly and heated at 180 °C for 24 h. The product was washed with de-ionized water 3 times and collected by centrifugation.

For comparison, other samples with different compositions were also synthesized by simply changing the addition quantity of Zn(Ac)_2_ to 0.16 g (molar ratio of Ag:Zn = 1:2), 0.4 g (molar ratio of Ag:Zn = 1:5), 0.04 g (molar ratio of Ag:Zn = 2:1) and 0.016 g (molar ratio of Ag:Zn = 5:1).

### 3.5. Characterization

The crystalline phases of the samples were analyzed by X-ray powder diffraction (XRD) (PANalytical B.V., Almelo, The Netherlands) on a Philips X’Pert diffractometer with Cu Kα radiation (λ = 0.15418 nm). The morphologies and sizes were observed with a Hitachi S-4800 field emission scanning electronic microscopy (FESEM) (Tokyo, Japan). Transmission electron micrographs (TEM) were obtained using a JEM-2100UHR transmission microscope (JEOL, Tokyo, Japan). The optical absorption spectra were measured on a UV-vis spectrometer (Shimadu UV2600, Kyoto, Japan) over a range of 200 to1000 nm.

### 3.6. Evaluation of Photo-Electrochemical Performance

For photo-electrochemical measurements, AgInS_2_-*x*Ag_2_S-*y*ZnS-*z*In_6_S_7_ heterojunctions were fabricated as the film electrodes. Typically, a glass substrate (ITO, 15 Ω/m^2^) was ultrasonically treated in a mixture of H_2_O, isopropanol, and acetone for 30 min. Then 30 mg of the as-synthesized samples and PEG aqueous solution (150 mg/mL) were mixed homogeneously. The obtained paste was spread onto the conducting glass substrate with a blade. Adhesive tapes were used as spacers. Finally, the resulted films with ~1 cm^2^ active area were annealed at 30 °C for 30 min under N_2_ atmosphere in order to achieve intimate contact between particles.

Photoelectrical response and electrochemical impedance spectroscopy (EIS) were measured on an electrochemical workstation (CHI 660E, CH Instruments, Chenhua, Shanghai, China) with a three-electrode cell. A Pt plate and saturated calomel electrode were used as the counter and reference electrodes, respectively. The as-prepared samples were employed as the working electrode with an active area of 1 × 1 cm^2^. The photocurrents were measured with the same three-electrode system under irradiation of a 300 W Xe lamp in 0.5 mol/L Na_2_SO_4_ aqueous solution. EIS was carried out with an open circuit potential (0.228 eV) from 1000 KHz–0.01 Hz in 0.50 M Na_2_SO_4_ containing equimolar [Fe(CN)_6_]^3−^ and [Fe(CN)_6_]^4−^ (2.5/2.5 mM) solution.

### 3.7. Evaluation of Photocatalytic Performance

The photocatalytic activity of the as-synthesized AgInS_2_-*x*Ag_2_S-*y*ZnS-*z*In_6_S_7_ nanocomposites was evaluated by the photocatalytic reduction of water to produce H_2_ under simulated sunlight irradiation. The H_2_ evolution test was performed on a LabSolar-III AG reaction cell (Beijing Perfect Light Company, Beijing, China). In a typical experiment, 20 mg of AgInS_2_-*x*Ag_2_S-*y*ZnS-*z*In_6_S_7_ nanoparticles were dispersed in an 60 mL of aqueous solution containing 2.6 g of Na_2_SO_3_ (0.33 mol∙L^−1^), 3.6 g of Na_2_S (0.74 mol∙L^−1^) as sacrificial reagents and 5 mL of 1 mg/mL Pt(NH_4_)_2_Cl_6_ as promoter. The aqueous solution was irradiated by a 300 W Xe arc lamp (PLS-SEX300/300UV, Beijing Perfect Light Company) to produce H_2_. The yield of H_2_ was determined with an on-line gas chromatograph (GC7900, Techcomp, Shanghai, China), which equipped with a molecular sieve 5A column and a thermal conductivity detector. N_2_ was used as the carrier gas.

## 4. Conclusions

In summary, we have synthesized a class of efficient solar-driven nanophotocatalysts made of AgInS_2_-*x*Ag_2_S-*y*ZnS-*z*In_6_S_7_ composite, where the compositions can be controlled by varying the feed ratio of Ag^+^ to Zn^2+^ ions. The solar hydrogen evolution tests show that the as-synthesized AgInS_2_-Ag_2_S-ZnS obtained with Ag^+^:Zn^2+^ of 1:1 exhibits the highest activity with a rate of 5.4 mmol h^−1^·g^−1^. Compared with the pristine AgInS_2_, AgInS_2_-Ag_2_S-ZnS shows a significantly enhanced photocatalytic H_2_ production performance by a factor of 14 and even higher than AgInS_2_-Ag_2_S-ZnS-In_6_S_7_ under the same conditions. The results demonstrate that AgInS_2_ nanoparticles integrated with appropriate proportion of Ag_2_S and ZnS can be used as a promising photocatalyst for H_2_ production.

## Supplementary Materials

The following are available online at www.mdpi.com/1996-1944/9/5/329/s1. Figure S1: Diffuse reflectance spectra of the as-prepared composite photocatalyhst with different ratio of Ag:Zn. Figure S2: The H_2_ evolved from water under solar light over AgInS_2_-Ag_2_S-ZnS obtained with Ag:Zn = 1:1. Figure S3: Photocatalytic H_2_ evolution under solar-light irradiation with the same sample as in Figure S2. It is showing that the addition of (NH_4_)_2_PtCl_6_ improved the production rate of H_2_ though the original catalyst also shows the good activity towards H_2_ evolution. Figure S4: BET surface area and H_2_ production of AgInS_2_-*x*Ag_2_S-*y*ZnS-*z*In_6_S_7_ with different molar ratios of Ag^+^ to Zn^2+^ ions. Figure S5: An SEM image of the AgI precursor.
