# Supplementary material for: Synthesis of AgInS2-xAg2S-yZnS-zIn6S7 (x, y, z = 0, or 1) Nanocomposites with Composition-Dependent Activity towards Solar Hydrogen Evolution
Source: Materials (Basel). 2016 Apr 29;9(5):329. doi: 10.3390/ma9050329 (PMC5503016; doi:10.3390/ma9050329)
Supplement: Supplementary file 1 [file materials-09-00329-s001.pdf]

Supplementary Materials: Synthesis of $AgInS_2$-$x Ag_2S$-$y ZnS$-$z In_6S_7$ (x, y, z = 0, or 1) Nanocomposites with Composition-Dependent Activity towards Solar Hydrogen Evolution

Zhaojie Wang, Shutao Wang, Junxue Liu, Wen Jiang, Yan Zhou, Changhua An and Jun Zhang

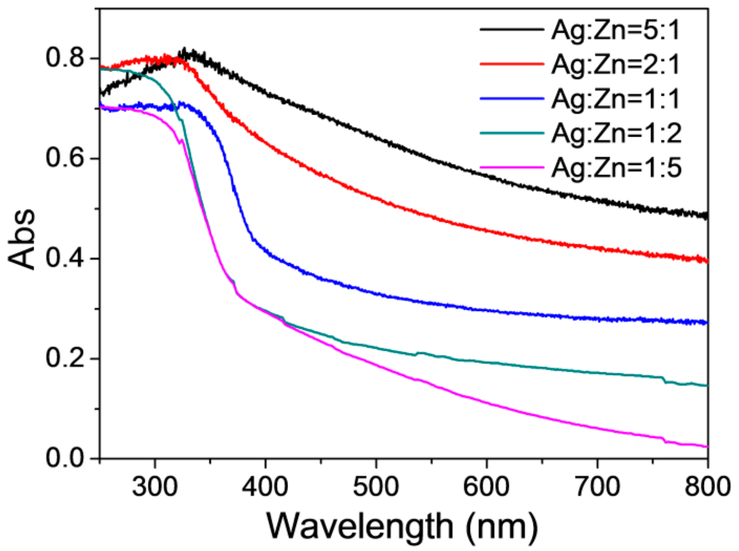

Figure S1. Diffuse reflectance spectra of the as-prepared composite photocatalyhst with different ratio of Ag:Zn.

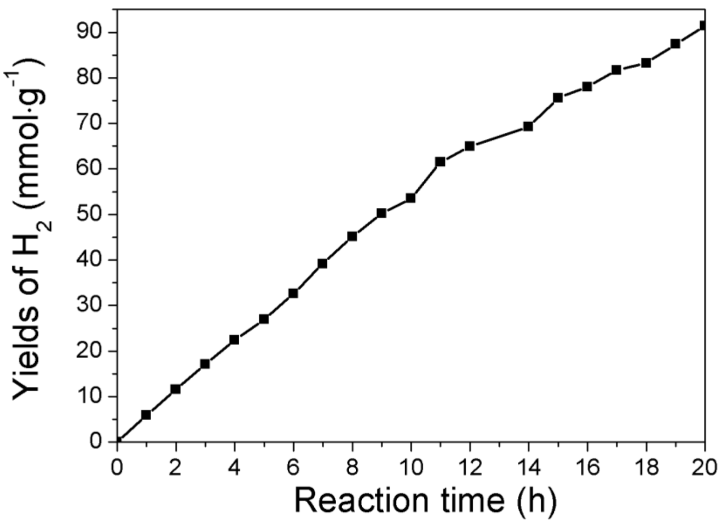

Figure S2. The H_2 evolved from water under solar light over $AgInS_2$-Ag_2S-ZnS obtained with Ag:Zn = 1:1.

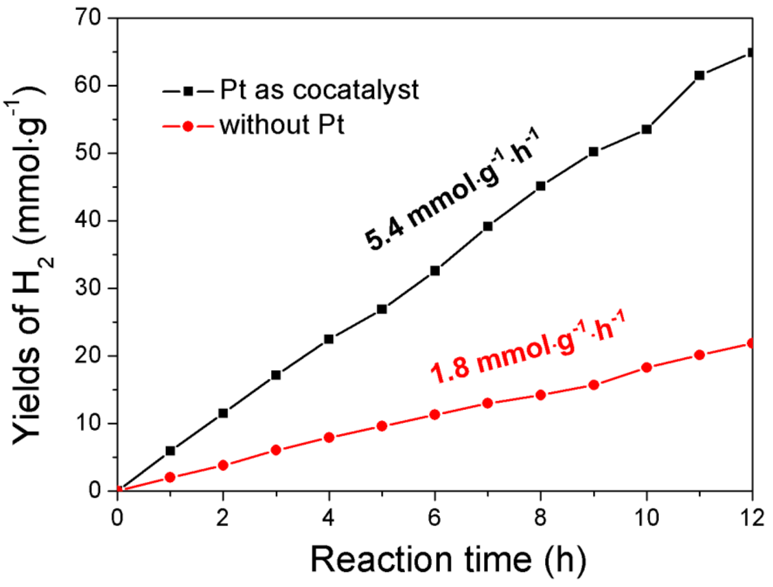

Figure S3. Photocatalytic H_2 evolution behavior under solar-light irradiation based on $AgInS_2$-Ag_2S-ZnS with or without Pt as cocatalyst. It showed that the addition of $(NH_4)_2PtCl_6$ improved the production rate of H_2.

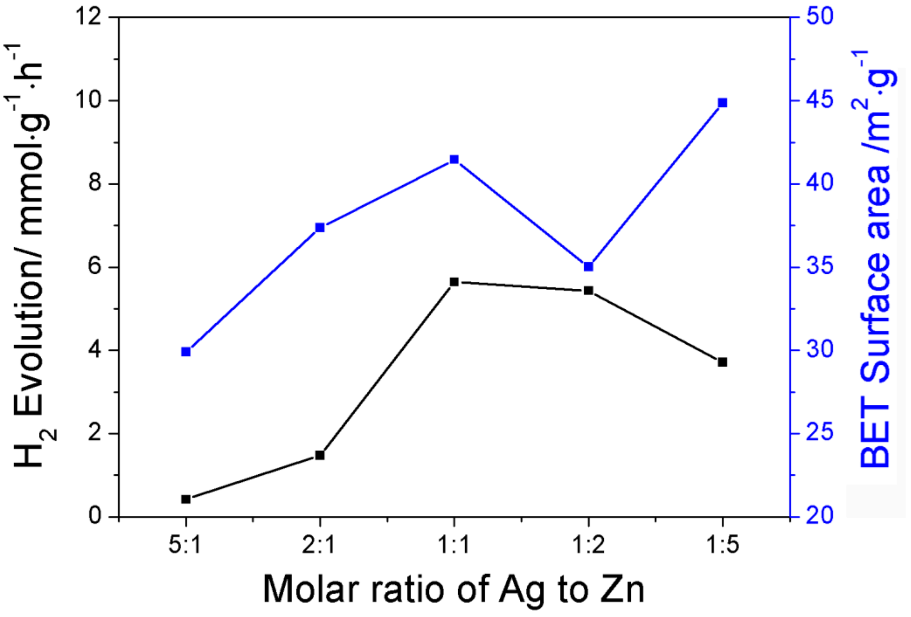

Figure S4. BET surface area and H_2 production of $AgInS_2$-$x$$Ag_2S$-$y$ZnS-$z$$In_6S_7$ with different molar ratios of Ag^+ to Zn^{2+} ions.

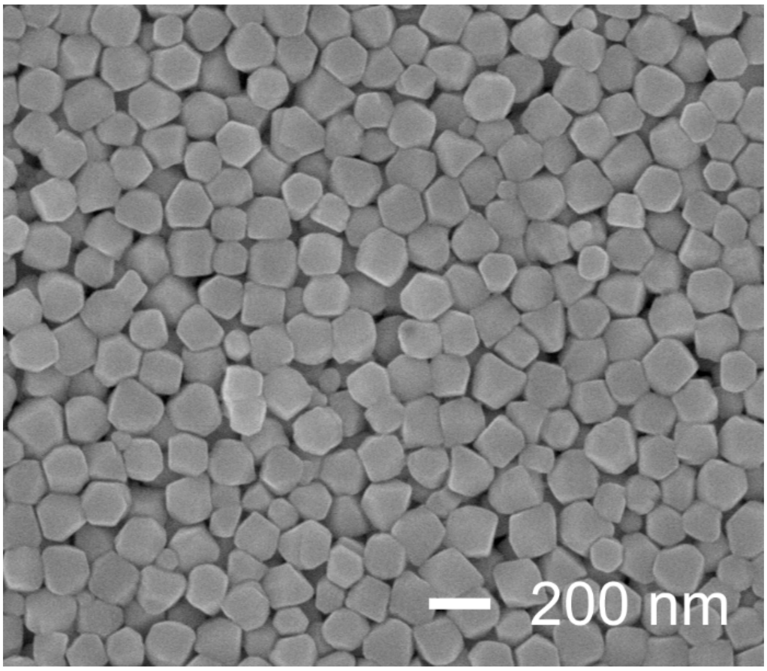

Figure S5. A scanning electronic microscope image of the AgI precursor.

Table S1. Comparison with other photocatalysts reported in the previous papers.

Photocatalyst	H_2 Evolution (mmol·g^{-1}·h^{-1})
Ag_2S·ZnS Core−Shell Heterostructures [1]	<0.25
ZnS-$CuInS_2$-$AgInS_2$ [2]	~7
ZnS-$CuInS_2$-$AgInS_2$ [3]	0.005
$AgInS_2$-ZnS [4]	3.15
$CuInS_2$-ZnS [5]	2.28
$AgInS_2$-Ag_2S-ZnS in our work	5.4

References

1. Zhu, T.; Zhang, C.; Ho, G. *In Situ* Dissolution−Diffusion toward Homogeneous Multiphase Ag/Ag2S@ZnS Core−Shell Heterostructures for Enhanced Photocatalytic Performance. *J. Phys. Chem. C* **2015**, *119*, 1667–1675, doi:10.1021/jp510413b.
2. Tsuji, I.; Kato, H.; Kudo, A. Visible-Light-Induced H_2 Evolution from an Aqueous Solution Containing Sulfide and Sulfite over a ZnS-$CuInS_2$-$AgInS_2$ Solid-Solution Photocatalyst. *Angew. Chem.* **2005**, *117*, 3631–3634, doi:10.1002/ange.200500314.
3. Tsuji, I.; Kato, H.; Kudo, A. Photocatalytic Hydrogen Evolution on ZnS-$CuInS_2$-$AgInS_2$ Solid Solution Photocatalysts with Wide Visible Light Absorption Bands. *Chem. Mater.* **2006**, *18*, 1969–1975, doi:10.1021/cm0527017.
4. Tsuji, I.; Kato, H.; Kobayashi, H.; Kudo, A. Photocatalytic H_2 Evolution Reaction from Aqueous Solutions over Band Structure-Controlled $(AgIn)_xZn_{2(1-x)}S_2$ Solid Solution Photocatalysts with Visible-Light Response and Their Surface Nanostructures. *J. Am. Chem. Soc.* **2004**, *126*, 13406–13413, doi:10.1021/ja048296m.
5. Tsuji, I.; Kato, H.; Kobayashi, H.; Kudo, A. Photocatalytic H_2 Evolution under Visible-Light Irradiation over Band-Structure-Controlled $(CuIn)_xZn_{2(1-x)}S_2$ Solid Solutions. *J. Phys. Chem. B* **2005**, *109*, 7323–7329, doi:10.1021/jp044722e.
